# CA-125 elimination rate constant K (KELIM) as a promising predictor of complete cytoreduction after neoadjuvant chemotherapy in advanced ovarian cancer patients: a retrospective study from two Chinese hospitals

**DOI:** 10.1186/s12885-024-12252-3

**Published:** 2024-05-20

**Authors:** Cheng Li, Qiulin Cui, Xuanhui Wang, Shuzhong Yao, Hua Tu, Ming Chen

**Affiliations:** 1 Department of Gynaecology, Guangdong Provincial Clinical Research Center for Obstetrical and Gynecological Diseases, The First Affiliated Hospital of Sun Yat-sen University, Zhongshan Second Road 1, 510080 Guangzhou, Guangdong China; 2 Department of Gynecologic Oncology, State Key Laboratory of Oncology in South China, Collaborative Innovation Center for Cancer Medicine, Sun Yat-sen University Cancer Center, Guangzhou, China

**Keywords:** Ovarian cancer, Neoadjuvant chemotherapy, Interval debulking surgery, CA-125 KELIM, Survival

## Abstract

**Background:**

The modeled CA-125 elimination constant K (KELIM) is a potential marker of tumor chemosensitivity in ovarian cancer patients treated with neoadjuvant chemotherapy (NACT) before interval surgery. The objective of this study was to externally validate the KELIM (rate of elimination of CA-125) score in patients with high-grade serous ovarian cancer (HGSC) undergoing NACT and explore its relation to the completeness of IDS and survival.

**Methods:**

The study was based on a retrospective cohort of 133 patients treated for advanced HGSC, International Federation of Gynecology and Obstetrics (FIGO) stages III–IV, with neoadjuvant chemotherapy, folllowed by interval surgery, in two centres in China. CA-125 concentrations at baseline and during neoadjuvant chemotherapy were collected. We used standardized (std) KELIM for subsequent analysis. Clinicopathologic parameters were collected, and Kaplan‒Meier survival analyses were performed for PFS and OS.

**Results:**

KELIM was an independent predictor of the probability of complete surgery and survival in our cohort. The median std KELIM score of patients with complete surgery was significantly higher than that of patients with incomplete IDS (1.20 vs. 0.71, *P* < 0.001). Multivariate analysis showed that a std KELIM score $\ge$0.925 was an independent predictive factor for achieving complete resection (OR = 5.480; 95% CI, 2.409–12.466, *P* < 0.001) and better PFS (HR = 0.544; 95% CI: 0.349–0.849, *P* = 0.007) and OS (HR = 0.484; 95% CI: 0.251–0.930, *P* = 0.030).

**Conclusions:**

The tumor-primary tumor chemosensitivity, assessed by the modeled CA-125 KELIM, calculated during NACT, is a major parameter to consider for decision-making regarding IDS attempts and predicting patient survival.

## Background

Ovarian cancer, the fourth most common cause of cancer-related death among women, is diagnosed in 240,000 women globally every year [1]. Traditionally, primary debulking surgery (PDS) followed by platinum-based chemotherapy is considered the standard treatment for advanced ovarian cancer. However, there are still some patients who could not benefit from this treatment. The National Comprehensive Cancer Network (NCCN) recommends neoadjuvant chemotherapy (NACT) followed by interval debulking surgery (IDS) as a standard treatment option for patients with a low probability of complete cytoreduction after PDS or who are unable to undergo surgery because of medical problems [2]. The goal of IDS should be complete resection of the tumor (R0 resection). Therefore, preoperatively identifying those patients who will respond to NACT and might reach complete cytoreduction is of utmost importance.

To more precisely quantify the extent of tumor load after NACT, multiple predictive models and ranking systems have been proposed [3–7]. However, no universally accepted imaging reference standard exists for the prediction of the completeness of surgery in HGSC. There is an urgent need for accurate, noninvasive predictors of the likelihood of complete cytoreduction and outcomes in patients with NACT to guide treatment decisions before preoperative patient selection.

Several blood-based markers have been proposed to predict prognosis in ovarian cancer, with CA-125 being the most widely known follow-up marker. The predictive values of CA-125 decline percentages during treatments were extensively investigated, with inconsistent outcomes [8–12]. More recent approaches based on artificial intelligence and mathematical modelling are promising strategies to define the equations describing longitudinal serum tumor marker kinetics during treatment and to subsequently extract modelled kinetic parameters expected to exhibit predictive values regarding treatment efficacy. The CA-125 KELIM (meaning CA-125 ELIMination rate constant K) is calculated with ≥ 3 CA-125 measurements during the first 100 days of chemotherapy, aiming to predict tumor chemosensitivity. In a study by You et al., the KELIM score was an independent prognostic factor for complete cytoreduction at interval surgery in both univariable and multivariable models [13]. Bouvarel et al.also validated the predictive value of the KELIM score for complete IDS and prognosis in their cohort just a few days ago [14]. Thus, the KELIM score may be a predictive marker to identify patients who will likely be poor responders to NACT and help clinical decision making regarding IDS and tailor adjuvant treatment in poor responders, including innovative therapies targeting platinum resistance.

The aim of this retrospective study was to investigate the efficacy of standardized (std) KELIM in HGSC patients receiving NACT and determine if std KELIM is predictive of complete resection at the time of IDS, PFS, OS; ultimately establishing its possible use as a pretherapeutic predictor of tumor chemosensitivity.

## Materials and methods

### Patients and clinical data

This study was designed as an observational, multicentre, retrospective study. We searched our electronic medical record systems for all advanced EOC patients who received NACT followed by IDS at the First Affiliated Hospital of Sun Yat-sen University between January 2015 and December 2022 and Sun Yat-sen University Cancer Center between January 2015 and December 2020.

The inclusion criteria were as follows: (1) patients pathologically confirmed as having FIGO stage III-IV primary high-grade serous ovarian cancer(HGSC); (2) treated with NACT followed by IDS consecutively in the First Affiliated Hospital of Sun Yat-sen University and Sun Yat-sen University Cancer Center; (3) patients with at least 1 abnormal CA-125 level (> 35 IU/ml) measured within 8 days before the first chemotherapy cycle and at least 3 CA-125 level determinations during NACT, allowing construction of a semimechanistic model of CA-125 kinetics; and (4) complete clinicopathological data.

Exclusion criteria were as follows: (1) other primary malignancies in other sites or second malignancies; (2) unavailable CA-125 level data or incomplete clinicopathological data; and (3) loss of follow-up data.

The following clinical data were collected: age, ECOG score, FIGO stage, pathology, cycles of NACT, CA-125 levels at diagnosis and prior to IDS, and HE4 levels at diagnosis and prior to IDS.

KELIM is the early modelling kinetic parameter in the mathematical modelling equation for longitudinal CA-125 kinetics during treatment. At least three available CA-125 values during the first 100 days of neoadjuvant chemotherapy were needed to calculate it. You et al. standardized KELIM to estimate KELIM’s predictive value for the possibility of complete IDS. As assessed in their previous studies, standardized (std) KELIM = KELIM/0.05. Std KELIM during NACT can be calculated at http://www.biomarker-kinetics.org/CA-125-neo [13, 15]. We used std KELIM for subsequent analysis.

All patients received 3–5 cycles of platinum and taxane-based NACT followed by IDS. During the study period, ovarian cancer patients received platinum-based chemotherapy (paclitaxel 175 mg/m2 and carboplatin AUC = 5) after surgery. Patients with advanced tumors were required to receive at least six cycles. At completion of the surgery, the completeness of the cytoreduction was estimated as follows: R0 resection (no residual disease), R1 resection (residual nodules $$ \le $$1 cm) and R2 resection (residual nodules $$ >$$1 cm). Complete cytoreductive surgery was defined as R0, whereas incomplete surgery included R1 + R2.

Both recurrence and progression after treatment are based on imaging evidence (CT or MRI). Treatment-free interval (TFI) was defined as the time interval between the date of the last chemotherapy session to the date of disease progression. Progression-free survival (PFS) and overall survival (OS) were defined as the time interval between the date of the primary surgery to the date of first recurrence and death/last contact, respectively.

### Follow-up

All patients were followed up after the end of adjuvant chemotherapy: every 3 months for the first 2 years, every 6 months for the next 3 years, and then annually thereafter. At each follow-up, patients’ status was assessed by tumor markers such as CA125, abdominal and pelvic ultrasound, CT, MRI or PET-CT as appropriate. The last follow-up date was Oct 10, 2023.

### Statistical analysis

The patient cohort was divided into a favorable group (patients who achieved R0 cytoreduction) and an unfavorable group (patients who didn’t achieve R0 cytoreduction) based on the surgical outcome of IDS. As baseline characteristics, continuous variables were reported as medians (range) or means (± standard deviation, SD), while categorical variables were reported as total numbers and percentages (%) appropriately. The differences in characteristics between the two groups were assessed by t test, Mann‒Whitney U test or Chi-squared tests. Receiver operating characteristic (ROC) curve analysis was performed to determine the predictive value of std KELIM and select an optimal cutoff point by Youden index [maximum (sensitivity + specificity-1)]. A multivariate logistic regression model was performed to assess the predictive value of variables regarding the likelihood of complete IDS. The Kaplan‒Meier method was used to construct survival curves, and the log-rank test was used to compare survival rates. Univariate and multivariate Cox regression models were performed to evaluate predictors of PFS and OS.

ROC curve analysis, survival analysis and all figures were performed and created by using R (4.2.3) software. Other tests were performed by using IBM SPSS Statistics version 25. A two-sided p value < 0.05 was considered statistically significant.

## Results

### Patient and clinical characteristics

**Fig. 1:**
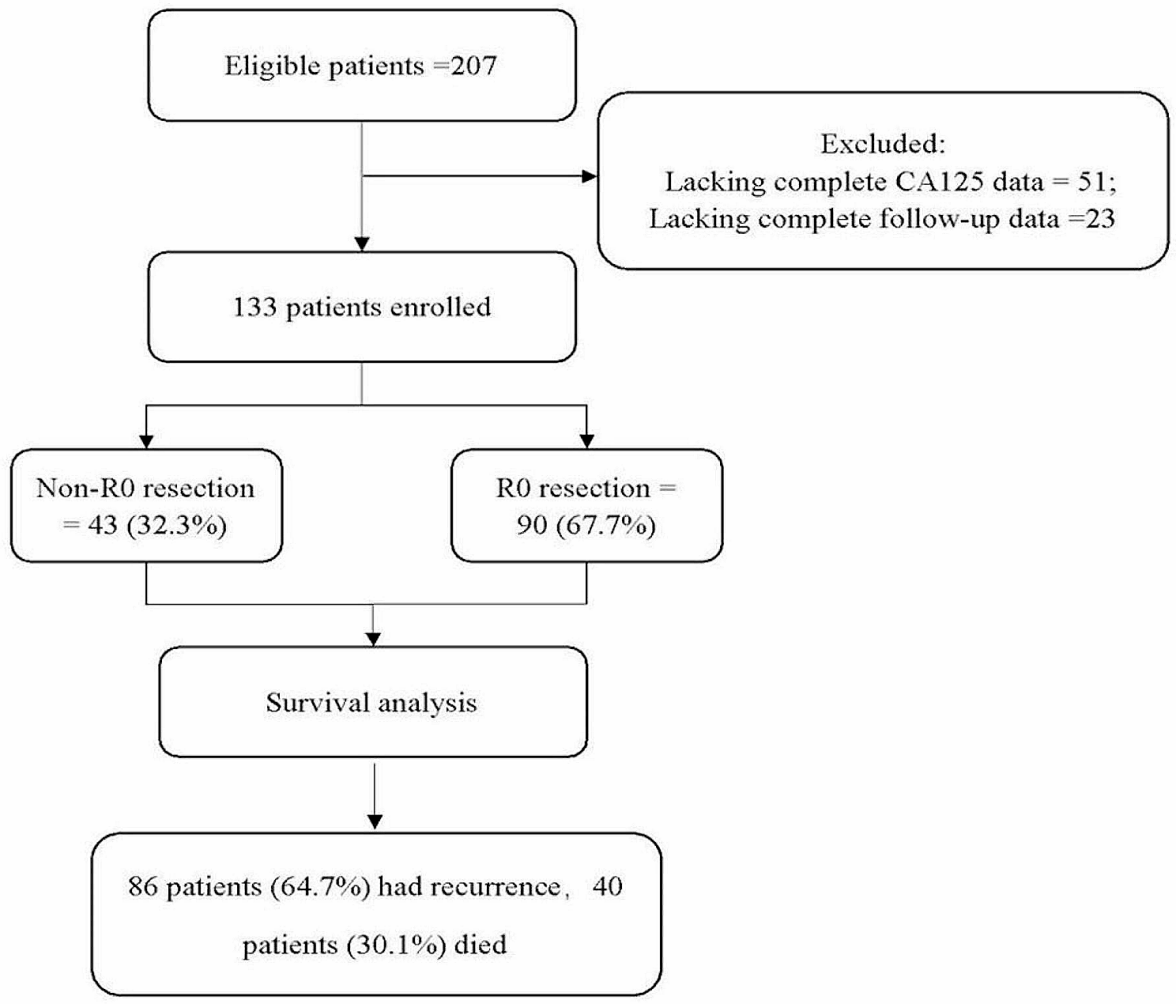
Flow chart illustratinging the patient inclusion

During the study period, 207 patient charts were reviewed, and 74 were excluded due to incomplete CA-125 variables during NACT or loss to follow-up (Fig. 1). The final cohort included 133 patients who met the inclusion criteria. The median follow-up time was 33 months (range, 6–80.8 months). Eighty-six patients (64.7%) had recurrence, and 40 patients (30.1%) died.

The demographic parameters of the study population are shown in Table 1. All of the patients had high-grade serous adenocarcinoma. Complete cytoreduction was achieved in 90 patients (67.7%). Among the 43 patients who had incomplete CRS, 13 patients received R2 resection surgery despite surgical effort due to massive carcinomatosis on the small bowel or basal pleura or on the main artery on the liver.

**Fig. 2:**
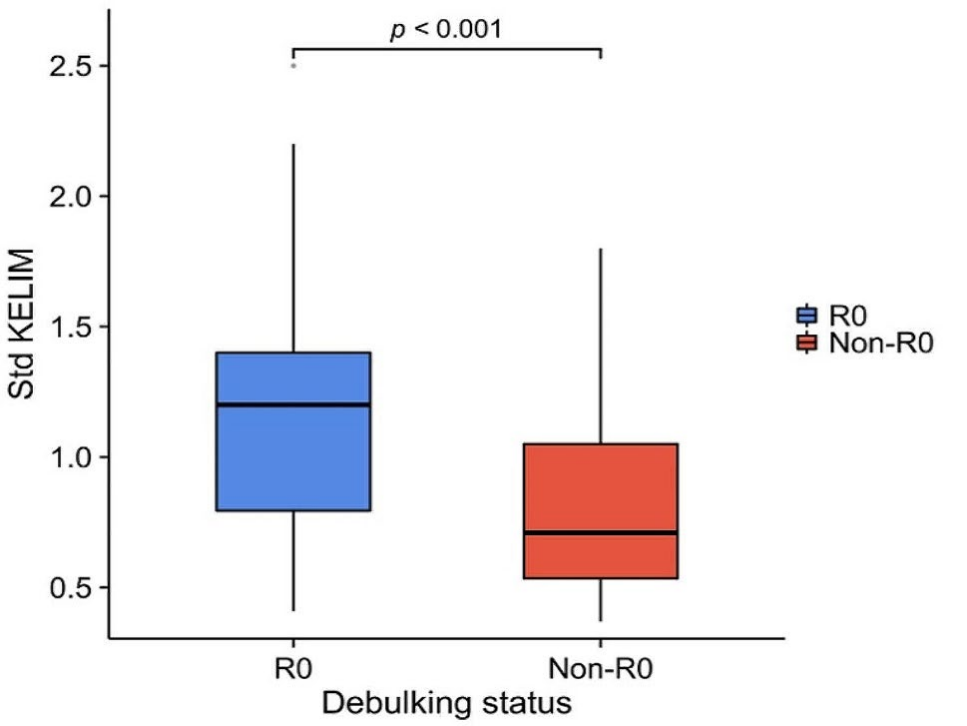
The distribution of standardized (std) KELIM scores in the R0 group and non-R0 group

There was no significant difference in age, ECOG score, FIGO stage, pathological type, cycles of NACT, CA125 levels at diagnosis or HE4 levels at diagnosis between patients who achieved R0 cytoreduction and non-R0 cytoreduction (*P* > 0.05). In the non-R0 group, patients had a lower median std KELIM score of 0.71 (range, 0.37–1.80; IQR, 0.58), whereas those with a complete CRS had a higher median std KELIM score of 1.20 (range, 0.41–2.50; IQR, 0.63) (*P* < 0.001, Table 1). The distribution of std KELIM scores in the two patient groups is shown by the boxplot (Fig. 2).

**Table 1:** Demographic parameters in patients with HGSC undergoing neoadjuvant chemotherapy

Characteristics	Overall population	R0	Non-R0	P-value
	(*n* = 133)	(*n* = 90)	(*n* = 43)	
**Age (Mean ± SD)**	56.4 ± 10	55.7 ± 9.8	57.8 ± 10.4	0.251
**ECOG (n, %)**				0.215
0–1	93(69.9)	66(73.3)	27(62.8)	
$\ge$2	40(30.1)	24(26.7)	16(37.2)	
**FIGO stage (n, %)**				0.337
Stage III	79(59.4)	56(62.2)	23(53.5)	
Stage IV	54(40.6)	34(37.8)	20(46.5)	
**Cycles of NACT [Median(range)]**	3(3,5)	3(3,5)	3(3,5)	0.178
**CA125 at diagnosis (U/mL) [Median(range)]**	1734.0(36.2,10000)	1672.6(36.2,10000)	2276.0(171.4,10000)	0.118
**HE4 at diagnosis (pmol/L) [Median(range)]**	619.3(3.5,6939.4)	577.4(3.5,5491)	722.2(44.1,6939.4)	0.437
**Std KELIM [Median(range)]**	1.00(0.37,2.50)	1.20(0.41,2.50)	0.71(0.37,1.80)	**<0.001**
**TFI (n, %)**				**<0.001**
<6 months	37(27.8)	16(13.3)	21(48.8)	
≥ 6 months	96(72.2)	74(86.7)	22(51.2)	
**Recurrence (n, %)**				**0.001**
Yes	86(64.7)	50(55.6)	36(83.7)	
No	47(35.3)	40(44.4)	7(16.3)	

### Association between std KELIM score and complete cytoreduction in the NACT population

**Fig. 3:**
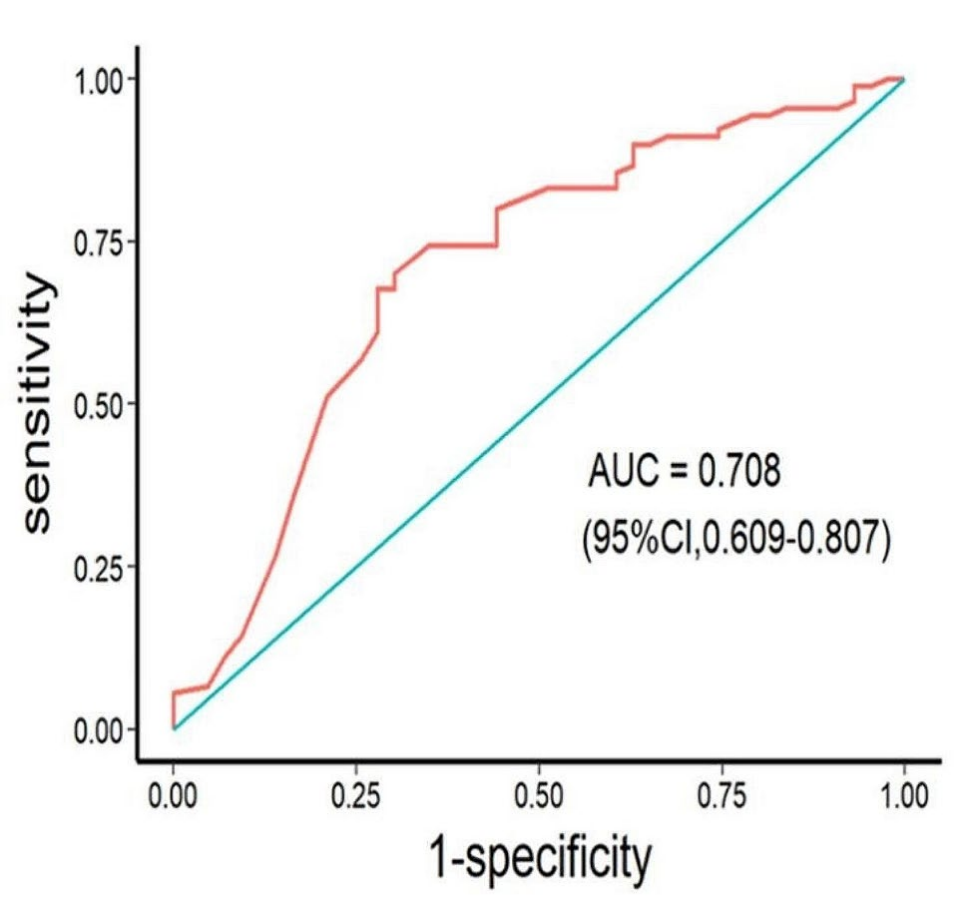
Receiver operating characteristic curve demonstrating the predictable value of standardized (std) KELIM for complete cytoreduction. (Abbreviations: AUC, area under the curve.)

Predictors for incomplete CRS were determined by ROCs (Table 2). According to these analyses, the best predictor of incomplete CRS was std KELIM, with an AUC of 0.708 (95% confidence interval [CI], 0.609–0.807). (Fig. 3). The maximum value of Youden index was 0.399, while sensitivity was 0.721 and specificity was 0.678. According to the Youden index, the cutoff point of std KELIM to predict complete cytoreduction was determined to be 0.925. As shown in Table 3, a std KELIM score $\ge$0.925 was validated as an independent predictive factor for achieving complete resection after multivariate analysis (OR = 5.480; 95% CI, 2.409–12.466, *P* < 0.001).

**Table 2:** Receiver operator curves (ROCs) for possible predictors of complete cytoreductive surgery

	P-value	AUC (95%CI)
ECOG score	0.479	0.538 (0.430–0.646)
FIGO stage	0.416	0.544 (0.438–0.649)
Age	0.240	0.563 (0.458–0.669)
Cycles of NACT	0.236	0.564 (0.459–0.668)
Std KELIM	**<0.001**	0.708 (0.609–0.807)

**Table 3:** Multivariate analysis regarding the likelihood of complete IDS (*N* = 133)

	OR	95% CI	P-value
**Age(year)**			0.648
< 56	REF		
$\ge$56	0.825	0.361–1.886	
**ECOG score**			0.445
0–1	REF		
$\ge$2	0.707	0.290–1.722	
**FIGO stage**			0.559
III	REF		
IV	1.281	0.557–2.945	
**Cycles of NACT**			0.125
3	REF		
>3	2.384	0.785–7.241	
**Std KELIM**			**<0.001**
< 0.925	REF		
$\ge$0.925	5.480	2.409–12.466	

### Association between std KELIM and survival in patients with IDS

**Fig. 4:**
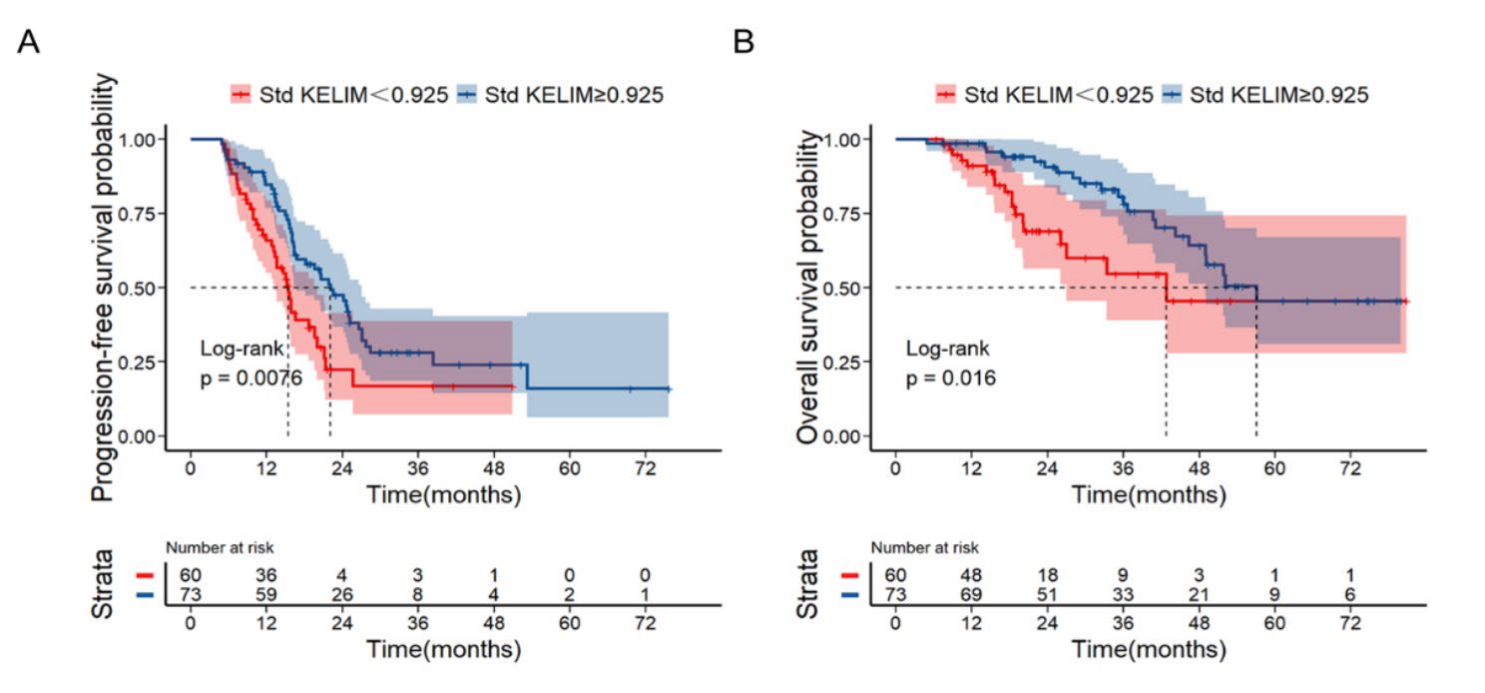
Kaplan-Meier curves of PFS **(A)** and OS **(B)** for patients according to standardized (std) KELIM (cut-off value = 0.925). (Abbreviations: PFS, progression-free survival; OS, overall survival.)

The median PFS and OS in the cohort were 18.1 and 51.8 months, respectively. In terms of survival, a std KELIM score $\ge$ 0.925 was associated with longer median PFS and OS (22.1 vs. 15.4 months, *P* = 0.0076, Figs. 4A and 57.1 vs. 42.8 months, *P* = 0.016, Fig. 4B) than a std KELIM score < 0.925.

In the multivariable analysis, after adjusting for age, ECOG score, stage, histology and NACT cycles, a std KELIM score $\ge$ 0.925 was an independent prognostic factor for PFS (HR = 0.544; 95% CI: 0.349–0.849, *P* = 0.007) and OS (HR = 0.484; 95% CI: 0.251–0.930, *P* = 0.030)(Table 4).

**Table 4:** Univariate and Multivariate analysis regarding for PFS and OS

Variables	PFS				OS			
	Univariate P value	Univariate HR(95%CI)	Multivariate P value	Multivariate HR(95%CI)	Univariate P value	Univariate HR(95%CI)	Multivariate P value	Multivariate HR(95%CI)
**Age(year)**	0.467		0.642		0.227		0.401	
< 56		REF		REF		REF		REF
$\ge$56		1.170(0.766–1.787)		1.112(0.712–1.737)		1.472(0.786–2.757)		1.322(0.690–2.532)
**ECOG score**	0.899		0.836		0.478		0.548	
0–1		REF		REF		REF		REF
> 1		1.032(0.638–1.669)		1.057(0.627–1.780)		1.300(0.629–2.684)		1.260(0.592–2.683)
**FIGO stage**	0.576		0.676		0.224		0.359	
III		REF		REF		REF		REF
IV		1.134(0.731–1.759)		1.104(0.694–1.755)		1.541(0.768–3.093)		1.403(0.680–2.892)
**Cycles of NACT**	0.523		0.391		0.508		0.509	
3		REF		REF		REF		REF
>3		0.825(0.457–1.490)		0.758(0.402–1.428)		0.745(0.312–1.780)		0.738(0.300-1.817)
**Std KELIM**	**0.008**		**0.007**		**0.019**		**0.030**	
< 0.925		REF		REF		REF		REF
$\ge$0.925		0.550(0.354–0.855)		0.544(0.349–0.849)		0.460(0.241–0.878)		0.484(0.251–0.930)

## Discussion

KELIM is a strong and reproducible prognostic marker in ovarian cancer that was developed and verified using data from over 12,000 patients who were carefully chosen for several large randomized clinical trials [16, 17].While the calculation of KELIM is available online, previous studies of std KELIM had mainly focused on Caucasian cohorts. Our study is the first analysis to explore the role of the std KELIM score as a predictor of primary tumor chemosensitivity in NACT of newly diagnosed advanced HGSC in Asian population. We confirmed the predictive value of KELIM for the possibility of complete resection at the time of IDS in HGSC patients in China, as well as its prognostic value on PFS and OS. These results demonstrated that KELIM score was applicable to Asian population as well.

The complete cytoreduction rate was 67.7% in our cohort, which was consistent with the literatures [18, 19]. A second study involving 2868 patients from ICON7 and AGO-OVAR7 and 9 revealed that patients with a favorable KELIM had a significantly greater likelihood of achieving complete IDS in comparison to those with an unfavorable KELIM [20]. Our study also showed that the std KELIM score was a reliable biomarker in advanced ovarian cancer after NACT. The std KELIM score was significantly higher in those patients who achieved complete resection than in those who received incomplete resection (1.20 with complete surgery vs. 0.71 with incomplete surgery; *P* < 0.001).

The basis for KELIM being an indicator of the probability of complete surgery might reflect the fact that KELIM is strongly associated with tumor primary chemo-sensitivity. According to the results of the CHIVA randomized phase II trial, patients with favorable std KELIM had better PFS and OS, irrespective of the completeness of IDS [13]. These survival benefits with KELIM score were also observed in our present study. This suggests that platinum sensitivity has a great impact on survival [21]. In our study, the std KELIM score was associated with survival. A std KELIM score ≥ 0.925 was associated with longer median PFS and OS (PFS: 22.1 vs. 15.4 months, *P* = 0.0076; OS: 57.1 vs. 42.8 months, *P* = 0.016) than a KELIM score < 0.925. These results revealed that the biological characteristics of tumors and their sensitivity to chemotherapy may influence the feasibility of surgical resection and overall survival in patients with HGSC. Hence, patients with higher std KELIM scores may derive greater benefit from conventional cancer therapies, including platinum-based regimens and cytoreductive surgery. Conversely, individuals with lower KELIM scores may require novel strategies to enhance chemosensitivity and improve prognosis. Potential approaches for chemosensitization, such as dose optimization with fractionated dose-dense chemotherapy or combination therapies like bevacizumab and cell cycle checkpoint inhibitors, should be explored in this patient population [15, 22, 23].

During our data collection, we also evaluated the predictive value of HE4 and found it did not correlate with surgical outcome, which was consistent with the literatures [24, 25]. HE4 levels are easily influenced by many physiological factors, such as the menopausal status, age, glomerular filtration capacity, smoking habit and body mass index. These factors may lead to its poor predictive ability in ovarian cancer [26].

This study is a retrospective study with inherent biases, including selection bias and information bias. Although we enrolled patients in two large cancer centres, the sample size was limited since some patients with incomplete CA125 data during chemotherapy and incomplete survival data were excluded. Meanwhile, BRCA mutation status was only assessable in 39.1% of patients in our cohort. 19 patients carried a BRCA mutation (BRCA1, 14 patients; BRCA2, 3 patients; BRCA1 and BRCA2, 2 patients). The low rate of BRCA results was due to the period of inclusion when the gene detection was not routinely performed. Since the sample number was small, further exploration about the relationship of std KELIM and BRCA mutation was unable to carry out. We will enroll more patients to answer these questions in the future.

In conclusion, our study is the first analysis to confirm the role of the std KELIM score as a predictor of primary tumor chemosensitivity in NACT of newly diagnosed advanced HGSC in Asian population. We revealed that Chinese HGSC patients undergoing NACT with a std KELIM score ≥ 0.925 were more likely to achieve complete resection in IDS and have better PFS and OS in comparison to those with a std KELIM score < 0.925. The KELIM score can be a useful tool for predicting chemosensitivity, surgical outcomes and survival outcomes in such patients undergoing NACT and subsequent IDS and may assist in treatment decisions. Prospective cohort studies regarding the application of the KELIM score in ovarian carcinoma are needed.

## Data Availability

Data may be available upon request to the corresponding authors.

## References

[1] Jemal A, Siegel R, Ward E, Hao Y, Xu J, Murray T, Thun MJ (2008). Cancer statistics, 2008. Cancer J Clin.

[2] NCCN Clinical Practice Guidelines in Oncology. Ovarian Cancer Including Fallopian Tube Cancer and Primary Peritoneal Cancer (Version 2.2022) [cited 2022 Jul 22]. Available from: https://www.nccnorg/professionals/physician_gls/pdf/ovarianpdf.

[3] Aletti GD, Dowdy SC, Podratz KC, Cliby WA (2007). Relationship among surgical complexity, short-term morbidity, and overall survival in primary surgery for advanced ovarian cancer. Am J Obstet Gynecol.

[4] Eisenkop SM, Spirtos NM, Friedman RL, Lin W-CM, Pisani AL, Perticucci S (2003). Relative influences of tumor volume before surgery and the cytoreductive outcome on survival for patients with advanced ovarian cancer: a prospective study. Gynecol Oncol.

[5] Fagotti A, Ferrandina G, Fanfani F, Ercoli A, Lorusso D, Rossi M, Scambia G (2006). A laparoscopy-based score to Predict Surgical Outcome in patients with Advanced Ovarian Carcinoma: a pilot study. Ann Surg Oncol.

[6] Sugarbaker PHJK (1995). Prognostic features of 51 colorectal and 130 appendiceal cancer patients with peritoneal carcinomatosis treated by cytoreductive surgery and intraperitoneal chemotherapy. Ann Surg.

[7] Suidan RS, Ramirez PT, Sarasohn DM, Teitcher JB, Iyer RB, Zhou Q, Iasonos A, Denesopolis J, Zivanovic O, Long Roche KC (2017). A multicenter assessment of the ability of preoperative computed tomography scan and CA-125 to predict gross residual disease at primary debulking for advanced epithelial ovarian cancer. Gynecol Oncol.

[8] Pelissier A, Bonneau C, Chereau E, de La Motte Rouge T, Fourchotte V, Darai E, Rouzier R (2014). CA125 kinetic parameters predict optimal cytoreduction in patients with advanced epithelial ovarian cancer treated with neoadjuvant chemotherapy. Gynecol Oncol.

[9] Zeng J, Yin J, Song X, Jin Y, Li Y, Pan L (2016). Reduction of CA125 levels during Neoadjuvant Chemotherapy can predict cytoreduction to no visible residual disease in patients with Advanced Epithelial Ovarian Cancer, primary carcinoma of fallopian tube and peritoneal carcinoma. J Cancer.

[10] Zhang D, Jiang YX, Luo SJ, Zhou R, Jiang QX, Linghu H (2018). Serum CA125 levels predict outcome of interval debulking surgery after neoadjuvant chemotherapy in patients with advanced ovarian cancer. Clin Chim Acta.

[11] Bartl T, Karacs J, Kreuzinger C, Pfaffinger S, Kendler J, Ciocsirescu C, Wolf A, Reinthaller A, Meyer E, Brandstetter M, et al. Tumor growth rate estimates are independently predictive of Therapy Response and Survival in Recurrent High-Grade Serous Ovarian Cancer patients. Cancers (Basel). 2021;13(5). 10.3390/cancers13051076. 10.3390/cancers13051076 PMC7959281 33802395

[12] Karamouza E, Glasspool RM, Kelly C, Lewsley LA, Carty K, Kristensen GB, Ethier JL, Kagimura T, Yanaihara N, Cecere SC, et al. CA-125 early dynamics to predict overall survival in women with newly diagnosed Advanced Ovarian Cancer based on Meta-Analysis Data. Cancers (Basel). 2023;15(6). 10.3390/cancers15061823. 10.3390/cancers15061823 PMC10047009 36980708

[13] You B, Robelin P, Tod M, Louvet C, Lotz J-P, Abadie-Lacourtoisie S, Fabbro M, Desauw C, Bonichon-Lamichhane N, Kurtz J-E (2020). CA-125 ELIMination rate constant K (KELIM) is a marker of Chemosensitivity in patients with ovarian Cancer: results from the phase II CHIVA trial. Clin Cancer Res.

[14] Bouvarel B, Colomban O, Frenel JS, Loaec C, Bourgin C, Berton D, Freyer G, You B, Classe JM (2024). Clinical impact of CA-125 ELIMination rate constant K (KELIM) on surgical strategy in advanced serous ovarian cancer patients. Int J Gynecol Cancer.

[15] Olivier C, Andrew C, Adrian C, Iain AM, You B (2023). Benefit from fractionated dose dense chemotherapy in patients with poor prognostic ovarian cancer: ICON-8 trial. JCO Clin Cancer Inf.

[16] You B, Freyer G, Gonzalez-Martin A, Lheureux S, McNeish I, Penson RT, Pignata S, Pujade-Lauraine E. The role of the tumor primary chemosensitivity relative to the success of the medical-surgical management in patients with advanced ovarian carcinomas. Cancer Treat Rev. 2021;100doi. 10.1016/j.ctrv.2021.102294. 10.1016/j.ctrv.2021.102294 34564042

[17] Lauby A, Colomban O, Corbaux P, Peron J, Van Wagensveld L, Gertych W, Bakrin N, Descargues P, Lopez J, Kepenekian V, et al. The increasing prognostic and predictive roles of the Tumor Primary Chemosensitivity assessed by CA-125 elimination rate constant K (KELIM) in ovarian Cancer: a narrative review. Cancers (Basel). 2021;14(1). 10.3390/cancers14010098. 10.3390/cancers14010098 PMC8750686 35008262

[18] Patel A, Iyer P, Matsuzaki S, Matsuo K, Sood AK, Fleming ND. Emerging trends in Neoadjuvant Chemotherapy for Ovarian Cancer. Cancers (Basel). 2021;13(4). 10.3390/cancers13040626. 10.3390/cancers13040626 PMC7915369 33562443

[19] Di Donna MC, Cucinella G, Zaccaria G, Lo Re G, Crapanzano A, Salerno S, Giallombardo V, Sozzi G, Fagotti A, Scambia G, et al. Concordance of Radiological, laparoscopic and laparotomic scoring to Predict Complete Cytoreduction in Women with Advanced Ovarian Cancer. Cancers (Basel). 2023;15(2). 10.3390/cancers15020500. 10.3390/cancers15020500 PMC9856465 36672451

[20] Moore K, Oza A, Colombo N, Oaknin A, Scambia G, Lorusso D, Farias-Eisner R, Banerjee S, Murphy C, Tanyi J, et al. Early prediction of the platinum-resistant relapse risk using the CA125 modeled kinetic parameter KELIM: a pooled analysis of AGOOVAR 7 & 9; ICON 7. Ann Oncol. 2019;30. 10.1093/annonc/mdz250.

[21] May T, Oza AM (2020). Measurement Tool of Chemotherapy Sensitivity in Advanced Ovarian Cancer. Clin Cancer Res.

[22] You B, Purdy C, Copeland LJ, Swisher EM, Bookman MA, Fleming G, Coleman R, Randall LM, Tewari KS, Monk BJ (2022). Identification of patients with ovarian Cancer experiencing the Highest Benefit from Bevacizumab in the First-Line setting on the basis of their tumor-intrinsic chemosensitivity (KELIM): the GOG-0218 Validation Study. J Clin Oncol.

[23] Mirza MR, Coleman RL, Gonzalez-Martin A, Moore KN, Colombo N, Ray-Coquard I, Pignata S (2020). The forefront of ovarian cancer therapy: update on PARP inhibitors. Ann Oncol.

[24] Vallius T, Hynninen J, Auranen A, Carpen O, Matomaki J, Oksa S, Virtanen J, Grenman S (2014). Serum HE4 and CA125 as predictors of response and outcome during neoadjuvant chemotherapy of advanced high-grade serous ovarian cancer. Tumour Biol.

[25] Plotti F, Scaletta G, Capriglione S, Montera R, Luvero D, Lopez S, Gatti A, De Cicco Nardone C, Terranova C, Angioli R (2017). The role of HE4, a Novel Biomarker, in Predicting Optimal Cytoreduction after Neoadjuvant Chemotherapy in Advanced Ovarian Cancer. Int J Gynecol Cancer.

[26] Qu W, Li J, Duan P, Tang Z, Guo F, Chen H, Zhu X, Jiang S-W (2016). Physiopathological factors affecting the diagnostic value of serum HE4-test for gynecologic malignancies. Expert Rev Mol Diagn.

